# Effectiveness of two procedures for deploying a facilitated collaborative modeling implementation strategy—the PVS-PREDIAPS strategy—to optimize type 2 diabetes prevention in primary care: the PREDIAPS cluster randomized hybrid type II implementation trial

**DOI:** 10.1186/s13012-021-01127-x

**Published:** 2021-05-27

**Authors:** Alvaro Sanchez, Susana Pablo, Arturo Garcia-Alvarez, Silvia Dominguez, Gonzalo Grandes, on behalf of the PREDIAPS Group, Amaia Bengoetxea, Olga Galarza, Elsa Martínez, Itziar Zalduegi, Dorothea Chausson, Agurtzane Gorroño, Alicia Pollán, Marisol Bernabéu, Mª Yolanda Calvo, Ander Artiagoitia, Nerea Zaramillo, Lidia Gonzalez, Asier Aurrekoetexea, Jon Azkarate, María Muñoa, Mar Bilbao, Vicki Camineiro, Gonzalo Gómez de Iturriaga, Fernando Gago, Iciar Ochoa de Retana, Ana Zorrilla, Mª Luisa Gutiérrez, Jone Capetillo Serra, Mª. Nieves Lopez, Nekane Iguerregui, Dolores López, Maite Gastañaga, Antonia Flores, Marcos Pereda, Amaya González, Ana Castresana, Laura Saiz, Nerea Regulez, Estibaliz Peciña, Jasone De la Plaza, Lucía Irastoza, Jose Contreras, Idoia Etxebarria, Begoña Oleaga, Cristina Herrero, Nora Cabezón, Fátima Calvo, J. Manuel Llamazares, Mª. Ángeles Gutierrez, Monica Prieto, Concepción Estébanez, María José Cordovilla, Alicia Domínguez, Isabel Lázaro, Elena Resines, Yolanda Villalba, Begoña Ayerdi, Florencia Martín, Magdalena Presmanes, Floreal Crespo, Araceli Benito, Mª. Belén Molina, Mª. Mar García, Mª. Gracia Díaz, Mª. Luisa Rodriguez Ortiz de Zarate, Rebeca San Cristóbal, M. Zugazaga Prieto, Pedro Martínez, Mercedes Crespo, Estíbaliz Albitre, Adelina García-Roldán, Amaia García, Maite Castro, Iñaki Gorospe, Amelia V. Hernández, Maite López, Mirian Sainz, Irene Marín, María Jesús Aragón, Leire Ulayar, Encarnación Santamaría, Carmen Sánchez, Javier Bayo, Begoña Urkullu, Ana Inés Pereda, Mercedes Garcia, Pilar Blanco, Silvia Soler Valverde, Jose I. Atela, Hiart Trespalacios, Anabel Llarena, Verónica Ruiz, Begoña Cabieces, Concepción Ugarte, Guadalupe Icaza, Edurne Zubeldia, Idoia González, Ángeles Gayo, Itxaso Arévalo, Gloria Intxausti, Esther García, Teresa Sánchez, Igone Lobato, Noelia Fuente, Naiara Ortolachipi, Edelweiss Sánchez, Victoria Cosgaya, Ángeles Gayo, Arantza Azazeta, Patricia Zaballa, Ana Isabel Ramila, Teresa Rodeño, Inmaculada Rodríguez, Teresa Vázquez, Raquel Ruíz, Rosa Herrero, María Valvanera, Saioa Setién, Begoña Ruíz, Juan José Casas, Joana Clemente, Javier Amiama, Javier Angulo, Belén Aramendia, Soledad Asenjo, Mª. Sonia Mayoral, Remedios Oyarzun, Bergoi Calvar, Belinda Zulueta, Edurne Elola, Josu Egaña, Gemma Díaz, Mª. Ángeles Sola, Laura Balague, Mª. Ángeles Ganzarain, Arantxa Aramburu, Ana Mª. Guinea, Edurne Lizarazu, Inma Valverde, Nekane Arenas, Susana Alonso, Rosa Salaberria, Javier Merino, Mercedes Álvarez, Ester Lázaro, Juncal Izcara, Leire González, Ainhoa García Leunda, Idoia Sánchez, Esther Usandiaga, Eluska Yetano, Jaione Larrea, Inés Mendinueta, Asún Uria, Ana Belén Gaztañaga, Eva Mayo, Onintza Aranzadi, Eulalia Medina, Rosa González, Ione Gutiérrez, Arantxa Perez, Mª. Ángeles Izquierdo, Alejandro García, Ainhoa Ugarte, Mª. Teresa Zubeldia, Bingen Uriondo, Mª. Carmen Aranegui, Arantxa Mendiguren, Yolanda Fernández, Maite Zapirain, Mª. Jose Garín, Aitziber Ayerbe, Jon Urkia, Alvaro Sánchez, Josep Cortada, Esther Gorostiza, Susana Pablo, Heather Lynn Rogers, Arturo García-Alvarez, Gonzalo Grandes, Alicia Cortazar, Virginia Bellido, Patxi Ezkurra, Rafa Rotaetxe

**Affiliations:** Primary Care Research Unit of Bizkaia, Deputy Directorate of Healthcare Assistance, Basque Healthcare Service - Osakidetza, Biocruces Bizkaia Health Research Institute, Plaza Cruces s/n, E-48903 Barakaldo, Spain

**Keywords:** Interprofessional collaboration, Implementation strategy, Diabetes prevention, Primary healthcare

## Abstract

**Background:**

The most efficient procedures to engage and guide healthcare professionals in collaborative processes that seek to optimize practice are unknown. The PREDIAPS project aims to assess the effectiveness and feasibility of different procedures to perform a facilitated interprofessional collaborative process to optimize type 2 diabetes prevention in routine primary care.

**Methods:**

A type II hybrid cluster randomized implementation trial was conducted in nine primary care centers of the Basque Health Service. All centers received training on effective healthy lifestyle promotion. Headed by a local leader and an external facilitator, centers conducted a collaborative structured process—the PVS-PREDIAPS implementation strategy—to adapt the intervention and its implementation to their specific context. The centers were randomly allocated to one of two groups: one group applied the implementation strategy globally, promoting the cooperation of all health professionals from the beginning, and the other performed it sequentially, centered first on nurses, who later sought the pragmatic cooperation of physicians. The following patients were eligible for inclusion: all those aged ≥ 30 years old with at least one known cardiovascular risk factor and an impaired fasting glucose level (≥ 110-125 mg/dl) but without diabetes who attended centers during the study period. The main outcome measures concerned changes in type 2 diabetes prevention practice indicators after 12 months.

**Results:**

After 12 months, 3273 eligible patients at risk of type 2 diabetes had attended their family physician at least once, and of these, 490 (15%) have been addressed by assessing their healthy lifestyles in both comparison groups. The proportion of at-risk patients receiving a personalized prescription of lifestyle change was slightly higher (8.6%; range 13.5-5.9% vs 6.8%; range 7.2-5.8%) and 2.3 times more likely (95% CI for adjusted hazard ratio, 1.38-3.94) in the sequential than in the global centers, after 8 months of the intervention program implementation period. The probability of meeting the recommended levels of physical activity and fruit and vegetable intake were four- and threefold higher after the prescription of lifestyle change than only assessment and provision of advice. The procedure of engagement in and execution of the implementation strategy does not modify the effect of prescribing healthy habits (p interaction component of intervention by group, p > 0.05).

**Discussion:**

Our results show that the PVS-PREDIAPS implementation strategy manages to integrate interventions with proven efficacy in the prevention of type 2 diabetes in clinical practice in primary care. Further, they suggest that implementation outcomes were somewhat better with a sequential facilitated collaborative process focused on enhancing the autonomy and responsibility of nurses who subsequently seek a pragmatic cooperation of GPs.

**Trial registration:**

Clinicaltrials.gov identifier: NCT03254979. Registered 16 August 2017—retrospectively registered.

Contributions to the literature

- Interprofessional collaboration (IPC) between physicians (FP) and nurses is a possible strategy to improve evidence-based type 2 diabetes (T2D) primary prevention in primary care (PC), yet best procedures to enable effective IPC teams are unknown.
- The PREDIAPS study aims to provide scientific knowledge regarding successful IPC-based interventions by assessing the effectiveness of two procedures for deploying a structured facilitated collaborative strategy—the PVS-PREDIAPS implementation strategy—to improve T2D prevention in PC.
- An IPC procedure for deploying the PVS-PREDIAPS strategy focused on enhancing the autonomy and responsibility of nurses as the main agents providing preventive care, obtain better implementation results.

## Introduction

The efficacy on lifestyle interventions for diabetes prevention has been well-established in clinical trials [1–4]. Nonetheless, initiatives to translate these interventions to primary care (PC) settings, though showing promising results, have encountered multiple barriers and challenges that impede actual integration of lifestyle interventions into routine healthcare delivery [4–7]. In relation to this, the Basque DEPLAN project for preventing type 2 diabetes (T2D), a multicenter clinical trial carried out in 14 Basque Health Service (Osakidetza) PC centers, demonstrated that an intervention focused on the promotion healthy lifestyles—four educational group or individual sessions plus an annual follow-up visit and regular contacts mainly via telephone calls—in patients with a high risk of developing T2D achieved a 32% reduction in the incidence of T2D [7]. Demonstration of its efficacy has not, however, been followed by feasible and sustainable translation of the intervention to clinical practice, mainly due to the difficulty of achieving engagement and commitment among staff of PC centers in the context of time constraints, heavy workload, lack of incentives, and an overload of health service initiatives [7, 8]. Given this, research is being conducted into various strategies to improve the skills of health professionals to address these barriers and thereby achieve effective prevention of the onset of T2D [9, 10].

It has been suggested that multidisciplinary team working, specifically, interprofessional collaboration (IPC) between PC physicians and nurses is a possible strategy for achieving the desired quality outcomes in an effective and efficient manner in an integrated health system [11–13]. IPC is a process by which different health professional groups work together to positively impact care and is characterized by mutual respect and trust, open communication between individuals, and holding a shared vision of the goals and potential benefits of a given intervention, as well as joint decision-making by consensus on how the intervention should be implemented. While IPC has shown to improve clinical practice and health outcomes [11, 13–15], it can also impede implementation of change in organizations due to problems related to professional boundaries and authority, difficulties in sharing knowledge, and limited understanding of others’ roles and responsibilities, among other factors [16–19].

Interprofessional practice-based interventions involve the deployment of a tool, routine, process, or activity within the practice setting to improve interprofessional interaction (e.g., communication and coordination). In turn, this may improve how healthcare professionals work together and deliver healthcare, leading to improved health outcomes [13]. Specifically, engagement of PC physicians and nurses in a facilitated process of collaborative modeling seems to be a promising way to achieve an increase in the practice of healthy lifestyle promotion in the context of routine care in this setting [20, 21]. Further, active and participatory engagement of professionals in collaborative processes [12, 22], as a way to enhance adaptation of interventions to the local context and its determinants [23, 24], has shown success in optimizing clinical practice. Research carried out on interprofessional collaboration considers that the interactions of team members, around the distribution of functions and boundaries between professions, pivot on two main axes of “autonomy” and “collaboration” [25]. Although a high grade of collaboration that may include the interchangeability of roles and functions can help reduce the workload of the team members, it can also increase the conflicts due to differences in power, values, structures, education, and relationships between these professional categories in a context with few explicit incentives for collaboration [18, 19, 25]. On the contrary, it seems that autonomy, promoting the empowerment of certain team members can be an important element of optimal interprofessional team functioning and successful collaborative interactions [25]. More research is needed, however, to test the effectiveness of the range of different IPC interventions, and establish the best procedures for creating the aforementioned IPC and under which circumstances IPC interventions are successful in improving or changing practice or facilitating the implementation of evidence-based practices [13].

The objective of the PREDIAPS study [10] was to assess and compare the effectiveness of two procedures for engaging health professionals and deploying an implementation strategy—the PVS-PREDIAPS implementation strategy—with the goal of increasing adherence to recommended clinical practice in the primary prevention of T2D and promotion of healthy lifestyles in prediabetic patients consulting in PC. All centers, headed by a local leader and an external facilitator, perform a structured process to model and adapt the intervention and its implementation to their specific context. Specifically, professionals after receiving training in the clinical intervention, identify areas for improvement based in a local needs assessment, collaboratively map intervention actions, flows, procedures and responsible agents, and finally standardize the intervention program after several pilot testing cycles. The centers were randomly allocated to one of two groups: one applied this strategy globally, promoting the cooperation of all health professionals from the beginning; and the other performed it sequentially, centered first on nurses, who subsequently sought to obtain the pragmatic cooperation of physicians. It was hypothesized that the sequential strategy will be more effective for accelerating the adoption of the recommended clinical intervention and ensures that it is maintained due to two main reasons. First, because nurses are the main agents responsible for the provision of intensive healthy lifestyles promote actions within PHC. Second, because nurses within the sequential strategy will autonomously consolidate the implementation of the clinical intervention at the level of its establishment (versus emphasis on the cooperation and collaboration of professionals of all levels within the global strategy). Once the clinical intervention is activated, it will try to organize a pragmatic cooperation with family physicians to maximize the efficiency of the intervention program flows and procedures, in a context of clear differentiation of roles and tasks. No differences are expected regarding the effectiveness of the clinical intervention by compared procedure as the clinical intervention to be integrated within the routine context is the same for both groups.

Specific objectives:

1. To assess the effect of different procedures (sequential vs. global) for engaging health professionals and deploying the PVS-PREDIAPS implementation strategy for increasing the proportion of individuals in whom clinical practice recommendations regarding T2D screening are met, and the proportion of high risk patients exposed to an evidence-based clinical intervention (assessment of lifestyle habits, provision of personalized advice, prescription of lifestyle changes and follow-up) to prevent T2D.
2. To assess the potential clinical effectiveness of the clinical intervention for changing lifestyle behaviors (physical activity and fruit and vegetable intake) and whether the conducted procedure for engaging professionals and deploying the implementation strategy modify this effectiveness.

## Methods

### Design

The PREDIAPS trial is a multicenter type II hybrid implementation trial carried out in nine PC centers to investigate the feasibility and potential efficacy of two procedures for engaging professionals and developing a facilitated collaborative modeling strategy to optimize the prevention of T2D in PC through the promotion of healthy lifestyles in high-risk individuals [10]. The study protocol was approved by the Clinical Research Ethics Committee of the Basque Country (Ref. No.: 08/2015) and the protocol was registered on Clinicaltrials.gov (identifier: NCT03254979; August 21, 2017). The CONSORT extension for Cluster Trials was used to guide reporting of the present study.

### Participants

Nine primary health centers out of 12 invited to participate from across 4 integrated healthcare organizations in Osakidetza were included in the study after obtaining written consent and agreement to participate from the majority (> 51%) of the nursing staff of the center and a substantial proportion of the family physicians whose patients would be involved through the nurses. Across the 9 centers, 65 physicians and 69 nurses agreed to collaborate (70% of all the physicians and 82% of all the nurses assigned to these centers).

Based on clinical practice guidelines [1–3, 8] and the actions implicit in the process of implementation (needs assessment and prioritization, piloting, and final standardization of the local intervention program) [10], the centers decided that the following patients would be eligible to participate: all individuals at a high risk of developing T2D, defined as an impaired fasting glucose level and at least one other known cardiovascular risk factor, among all patients ≥ 30 years old, who sought medical attention at least once through the participating centers between March 15, 2017 and March 15, 2018, this being the program implementation period. During this program implementation period, centers decided to extend the program beyond the prevention of DM2 in high-risk patients, offering it to other possible patients who may benefit from a healthy lifestyle promotion (e.g., overweight or obese patients with normal glucose levels).

### Clinical intervention

Based on the scientific evidence and clinical practice guidelines available on T2D prevention [1–3, 8], it has been recommended that a system should be deployed for screening to identify high-risk individuals and those identified should be offered an intensive lifestyle intervention and monitored closely. Such individuals should do at least 150 min of moderate physical activity a week and follow a Mediterranean-type healthy or low-calorie low-fat diet. To prepare individuals for changing their lifestyle and achieve behavior change, it has been suggested that they should undergo assessment of whether they meet healthy lifestyle recommendations and receive personalized advice including a tailored lifestyle prescription setting attainable targets.

The PVS clinical intervention [26], grounded in evidence-based theoretical models explaining behavior change and structured following the 5 A’s (Ask, Advise, Agree, Assist, and Arrange follow-up) intervention framework [27], was used to standardize the provision of most of the evidence-based behavior modification techniques used to promote changes in the targeted lifestyles seeking to prevent T2D. The PVS clinical intervention is considered useful in PC for its feasibility as well as for the scientific evidence supporting its effectiveness [20, 21]. To favor the execution of the clinical intervention, a computer tool integrated in the electronic health record guides and helps healthcare professionals to: (a) assess the lifestyle behaviors of patients and their compliance with current lifestyle-related recommendations; (b) provide personalized medical advice tailored to the patient and prescribe lifestyle change with plans for modifying physical activity or dietary habits; and (c) register and store the data on the actions carried out in each case to facilitate monitoring [20, 26].

### Implementation strategy

**Fig. 1:**
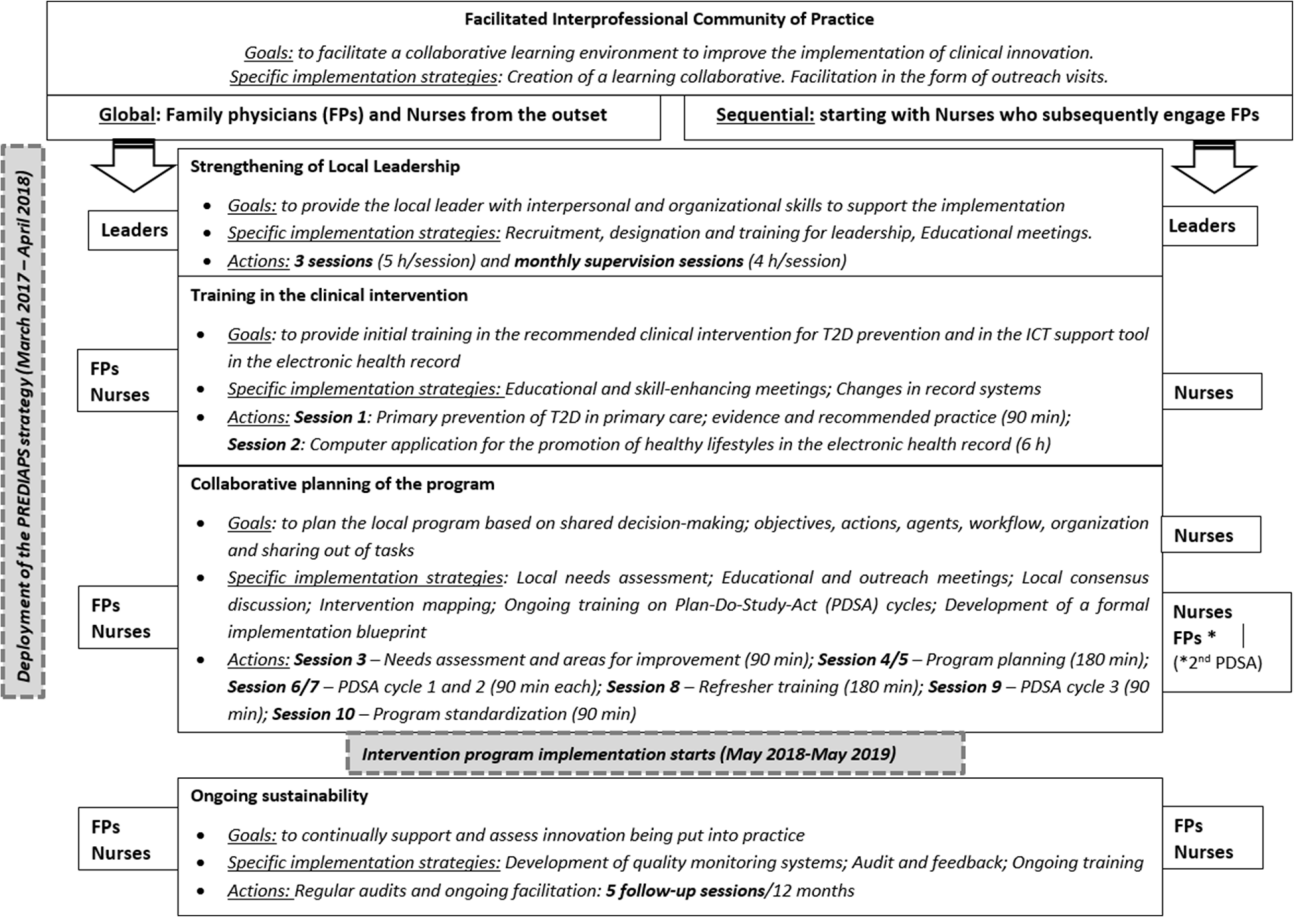
Compared procedures for engagement of professionals and deployment of the PVS-PREDIAPS implementation strategy

The PVS-PREDIAPS implementation strategy is based on a collaborative modeling process with an external facilitator, allowing the evidence-based clinical intervention for the primary prevention of T2D through the promotion of healthy lifestyles to be adapted to the context and determinants of local clinical practice of the centers, maximizing the feasibility of performing the intervention and its efficacy in real-world clinical practice conditions [10, 28]. The strategy, based on the creation of interprofessional community of practice led and driven by a previously selected local leader and an external facilitator, has an implementation phase with three key components and a post-implementation phase. These components, through which the specific implementation actions and strategies are operationalized and put into practice are (a) promotion of local leadership through the identification, initial training, and ongoing support of local leaders throughout the implementation process and development of clinical intervention programs tailored to the local context; (b) technical training on the clinical intervention for the promotion of healthy lifestyles for T2D prevention and the IT tools designed to standardize and support delivery of the intervention; (c) planning, collaborative modeling of the local program for primary prevention of T2D, and undertaking of short pilot studies of specific actions to assess and optimize the efficacy, flows, and procedures of the intervention program; and (d) ongoing monitoring, support, and facilitation of the deployment of the program as part of the routine activity of the centers (see Fig. 1). A detailed description of the PVS-PREDIAPS implementation strategy and its actions has been published elsewhere [10, 28].

### Randomization

The health centers were randomly assigned to one of two groups with the same implementation strategy, but following different procedures, sequential, or global, in relation to the engagement of professionals and deployment of the implementation strategy. Specifically, four centers were allocated to perform the PVS-PREDIAPS strategy globally, that is, involving both physicians and nurses at the same time, from the outset, in carrying out the implementation strategy and associated actions, while the other five centers were allocated to the sequential procedure, in which initially only nurses are involved and, subsequently, it is these nurses who seek to engage physicians, in a pragmatic way, in a cooperative process [10]. To carry out the randomization, a technical member of staff, independent of the study, produced a computer-generated random number sequence. As the clinical intervention for T2D prevention itself is the same with both procedures for the engagement in and execution of the strategy, participating patients are expected to be blind to their group allocation.

### Fidelity of the implementation strategy

The execution of the implementation strategy and hence the type of interprofessional community of practice created differ between the groups in terms of the degree of exposure and collaborative participation of professionals in the following actions: initial training, planning, program modeling, and piloting prior to the standardization of the definitive program at the local level. Specifically, in the sequential strategy, physicians are less exposed to the strategy actions (4.5 h out of the total of 20 h), and hence, less involved in the collaborative work on the modeling and tailoring of the intervention program to the local context of the centers, this task mainly falling to nurses. The PVS-PREDIAPS implementation strategy to improve T2D primary prevention has been carried out with high degree of fidelity. Despite some differential exposure to the overall strategy within the nursing staff in the groups compared, the professionals involved have received notably high exposure to the implementation strategy, and the planned program differentiation (related to engagement of professionals and deployment of the implementation strategy) has been attained. The implementation strategy and fidelity of execution have been described in detail elsewhere [10, 28].

By carrying out the actions of the implementation strategies, the staff at the PC centers have designed and tailored a T2D prevention program, sharing the actions of the recommended clinical intervention among the professional groups involved. In general, physicians perform the screening and refer high-risk patients identified to nurses for delivery of the intervention promoting healthy lifestyle habits; the nurses first ask patients about their lifestyle and then provide personalized advice tailored to the patient’s needs, encouraging individuals motivated to make lifestyle changes to attend an additional consultation at which a lifestyle change is prescribed and a personalized plan for modifying habits and monitoring change achieved over time is developed in collaboration with the patient. The distribution of the components of the intervention is established at the level of PC team, however, and the clinicians are allowed to opt for a different approach (e.g., physicians asking about habits and providing advice, as well as screening for T2D risk) (see of models of prevention programs for T2D, Figure 6 in Appendix). During the implementation period, the clinical intervention was extended to other types of patients not meeting the criteria for a high risk of developing T2D (e.g., overweight or obese patients with normal glucose levels).

### Assessment of outcomes

Implementation indicators related to the execution of the clinical intervention and user sociodemographic (age, sex, socioeconomic status) and clinical characteristics were calculated from routine data extracted from the electronic health records of Osakidetza. The Primary Care Research Unit of Bizkaia is explicitly authorized by the Healthcare Management of the Basque Health System to extract and use data from the electronic health records for research purposes. These data are confidential, are processed anonymously, and used solely for the purpose of the study (Spanish Act 15/1999 of December 13 on the protection of personal data). The Primary Care Research Unit of Bizkaia is responsible for not only the handling of data and ensuring their quality but also for the coordination, process quality control, and execution of the study.

Specifically, within the clinical intervention program, healthy lifestyles were assessed with the 10-item PVS-Healthy Lifestyle Screening questionnaire, the validity of which has been demonstrated in a previous study [29]. A Deprivation Index (DI), defined by census tract, developed in 2008 [30] was used as a socioeconomic status indicator. This index is an ordinal variable, categorized into five levels (DI quintiles), providing a measure of the socioeconomic characteristics of the population of census tracts. It provides an estimate of socioeconomic and environmental inequalities among inhabitants by census tract in Spain. The calculation takes into account the percentages of residents in a tract who are manual workers, unemployed, temporary employees, or have a poor level of educational attainment, overall and also specifically among young people, based on the most recent census data available (2016).

#### Outcome measures

The RE-AIM (Reach, Efficacy, Adoption, Implementation, and Maintenance) as evaluation framework [31] was used to inform study’s main outcome indicators in order to facilitate the interpretation of results in terms of public health significance. Specifically, the following outcome indicators based in the RE-AIM dimensions are assessed:

##### Reach—T2D screening

T2D screening as part of opportunistic screening for cardiovascular risk in individuals aged ≥ 30 years with at least one known risk factor:

- % of non-diabetic patients aged ≥ 30 years with a cardiovascular risk factor (e.g., hypertension or body mass index [BMI] ≥ 30 kg/m^2^ or hyperglycemia) attending consultations with their family physician during the program implementation period (from March 2, 2017 to March 2, 2018) in whom clinical practice guidelines regarding T2D screening have been followed, namely, the patient has had at least one fasting glucose level test within the year before the date of attendance.
- % of non-diabetic patients with a cardiovascular risk factor (e.g., hypertension or BMI ≥ 30 kg/m^2^) attending their family physician considered at high risk of developing T2D as they have had a fasting glucose level of 110 to 125 mg/dl in at least one cardiovascular disease (CVD) or T2D screening test within the year before the date of attendance.

##### Reach—T2D prevention program

- % non-diabetic patients aged ≥ 30 years at high risk of developing T2D defined by the presence of a CVD risk factor and of prediabetes (fasting glucose 110 to 125 mg/dl in any CVD or T2D screening test within the year before the date of attendance or during the implementation period) in which at least one lifestyle behavior has been assessed.

##### Reach—spread

Healthy lifestyle promotion actions in attending patients who do not meet the criteria for a high risk of developing T2D (e.g., patients already diagnosed with diabetes, overweight, or obese patients with normal glucose levels)

- % of patients whose physical activity levels and/or diet have been assessed given preventative advice concerning the need to increase physical activity and/or eat a healthy diet; or prescribed lifestyle change with a plan for increasing physical activity and/or eating a healthy diet, among all those attending aged 10 to 80 years, 12 months after the setting up of the program.

##### Efficacy

The clinical outcome measure concerns to the potential effectiveness of the intervention among the groups compared, to ascertain whether the procedure of professional involvement and deployment of the PVS-PREDIAPS implementation strategy performed modifies the effect of the intervention. Specifically, the measures compared are:

- % of individuals who did not meet the recommendations for physical activity, and after the intervention, did at least 150 min of moderate or 75 vigorous physical activity per week.
- % of individuals who did not meet the recommendations on fruit and vegetable intake, and after the intervention, reported eating at least five portions a day.

An observational study was conducted of the effect of the intervention on changes in habits, stratifying by implementation strategy used, in an incidental sample of patients exposed to the healthy lifestyle promotion program for the prevention of T2D and other cardiovascular diseases. The sample was composed of patients who had their lifestyle habits assessed using the PVS questionnaire [29], at program inclusion and at least 9 months after exposure to the intervention as part of routine healthcare or the annual follow-up recommended by clinical guidelines for the primary prevention of T2D in high-risk patients.

##### Implementation

Execution of the components of the clinical intervention in non-diabetic patients aged ≥ 30 years at high risk of developing T2D attending their family physician during the program implementation period:

- % of patients whose physical activity levels and/or diet have been assessed, 12 months after the setting up of the program (reach of the prevention program for T2D)
- % of patients who have been given preventative advice concerning the need to increase physical activity and/or eat a healthy diet, 12 months after the setting up of the program
- % of patients who have been prescribed lifestyle change with a plan for increasing physical activity and/or eating a healthy diet, 12 months after the launch of the program

No safety analysis was performed as we did not anticipate any adverse effects associated with the intervention since it only involved promotion of a healthy lifestyle (minimum recommended levels of physical activity and a balanced diet), this being the intervention recommended for the prevention of T2D in high-risk patients [1–3, 8].

### Sample size

For sample size calculation for the primary outcome, being this the primary prevention practice actions for T2D prevention (e.g., assessment of healthy lifestyles, advice on changing lifestyles), we hypothesized an increase in the sequential group of 30% in the percentage of patients receiving any of the recommended T2D prevention actions over a rate of 50% obtained in the global group (that is, 65% in the sequential group compared to 50% in the global group). Considering that 9 centers participated, an alpha of 0.05, and an intraclass correlation at center level of 0.03, we estimated that we needed at least 425 individuals at high risk of developing T2D eligible to be exposed to prescription of lifestyle change (with personalized plans for modifying unhealthy habits) per group (850 in total) to achieve a statistical power of over 80%, assuming a loss to follow-up of 20%, to compare process indicators related to exposure and execution of the intervention.

### Statistical analysis

Frequencies and proportions were used to describe professional, center, and patient characteristics for continuous and categorical variables, respectively. Indicators related to the implementation of the recommended intensive intervention promoting healthy lifestyle habits for T2D prevention (rate of prescribing of a healthy lifestyle) and other components of the intervention program (for example, rate of screening for high risk of T2D by measuring fasting glucose levels in people ≥ 30 years old with known cardiovascular risk factors) were also expressed as frequencies and percentages for each collaborating center and by group. Survival analysis was used to compare the incidence of exposure to the intervention components (time until exposure to lifestyle assessment, advice, and prescription of lifestyle change) between the groups compared. For univariate analysis of the association of time until exposure to the different intervention components with the comparison groups, cumulative survival probabilities over 12 months were estimated and compared using Kaplan-Meier curves along with the log-rank and Wilcoxon tests. Time to event or censoring was defined as the time between study start date (May 2, 2017) and the date of exposure to the specific intervention component, or possible censoring at the end of the study (May 2, 2018), respectively. Adjusted hazard ratios (AHRs) and 95% confidence intervals (CIs) describing associations between group or patient characteristics and the exposure to the intervention components were estimated using Cox proportional hazards models. These models included PC center, sex, age, recorded chronic diseases, and the deprivation index as independent factors. The proportional hazard assumption was tested, introducing the interaction of each of the variables with time in the models. In the case of covariates for which the proportional hazard assumption was not satisfied, the Cox model was extended with time-dependent variables allowing hazard ratios to change over time.

With regards to the clinical outcome measures, the percentages (e.g., % of patients who meet the physical activity recommendations) are compared using chi-squared tests. Stratified analysis and adjusted mixed-effect generalized models were used to estimate the differences between groups adjusted for confounders and modifiers of the effect of the intervention (baseline demographic and clinical characteristics), and adjusted differences, adjusted odds ratios (AORs) and their 95% CIs were calculated at the patient level, taking into account the hierarchical and multicenter nature of the data with patients clustered by physician and physicians nested in centers. Treatment group, baseline values, and patient clinical and demographic characteristics were considered fixed effects. Centers and physicians were included as random effects on the intercept and on treatment effect. Likelihood ratio tests (significance threshold, P > .05) were used to simplify the models following a backward strategy. Finally, to assess potential modification of the effect of the intervention on clinical outcomes attributable to the procedure used for professional engagement in and deployment of the PVS-PREDIAPS implementation strategy, a term was introduced to represent the interaction between the dose of the intervention received (assessment of habits alone, assessment plus advice, assessment plus advice plus prescription) and the group in relation to the procedure carried out. All the analysis was performed with the SAS software.

## Results

### Results of the implementation by center and group

**Fig. 2:**
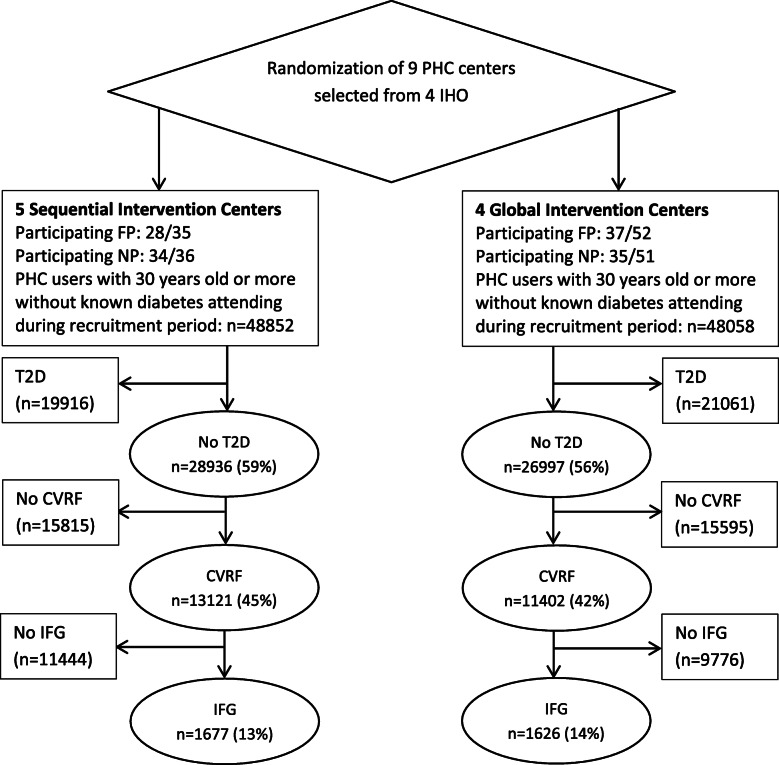
Flow of patients through the PREDIAPS study (following the CONSORT extension for cluster trials). Note: FP, family physician; IHO, integrated healthcare organization; NP, nurse practitioner; ≥ 30 years, patient aged ≥ 30 years that attended their family physician at least once; No T2D, patients without registered diagnosis of type 2 diabetes; CVRF, patients with a cardiovascular risk factor (e.g., overweight or obese patients with normal glucose levels); IFG, patients with impaired fasting glucose level at any visit during the program implementation period

After 12 months, a total of 26,997 and 28,936 non-diabetic patients aged ≥ 30 years old attended their family physician at least once in the global and sequential centers, respectively, this corresponding to 56% and 59% of patients ≥ 30 years old on the lists of the participating centers in each group; and of these, 11,332 and 13,031 had a known risk factor (e.g., hypertension) (43.6% of ≥ 30-year-olds seen at least once). Overall, 77.8% of these patients (18,944 of 24,363) had been screened for T2D by measurement of their fasting glucose level within the last year as recommended in clinical practice guidelines. Among the screened patients, 1626 (14.3%) and 1647 (12.6%) were eligible for program inclusion in the global and sequential groups, respectively, as they were aged ≥ 30 years old, and had an impaired fasting glucose level (≥ 110-125 mg/dl) and at least one other known CVD risk factor before any of their visits (see Fig. 2 Flow of the study following the CONSORT extension for cluster trials). Among the eligible patients at high risk of T2D, half were female and the mean age was 67 years. A slightly higher proportion of patients were overweight or obese in the sequential group.

**Fig. 3:**
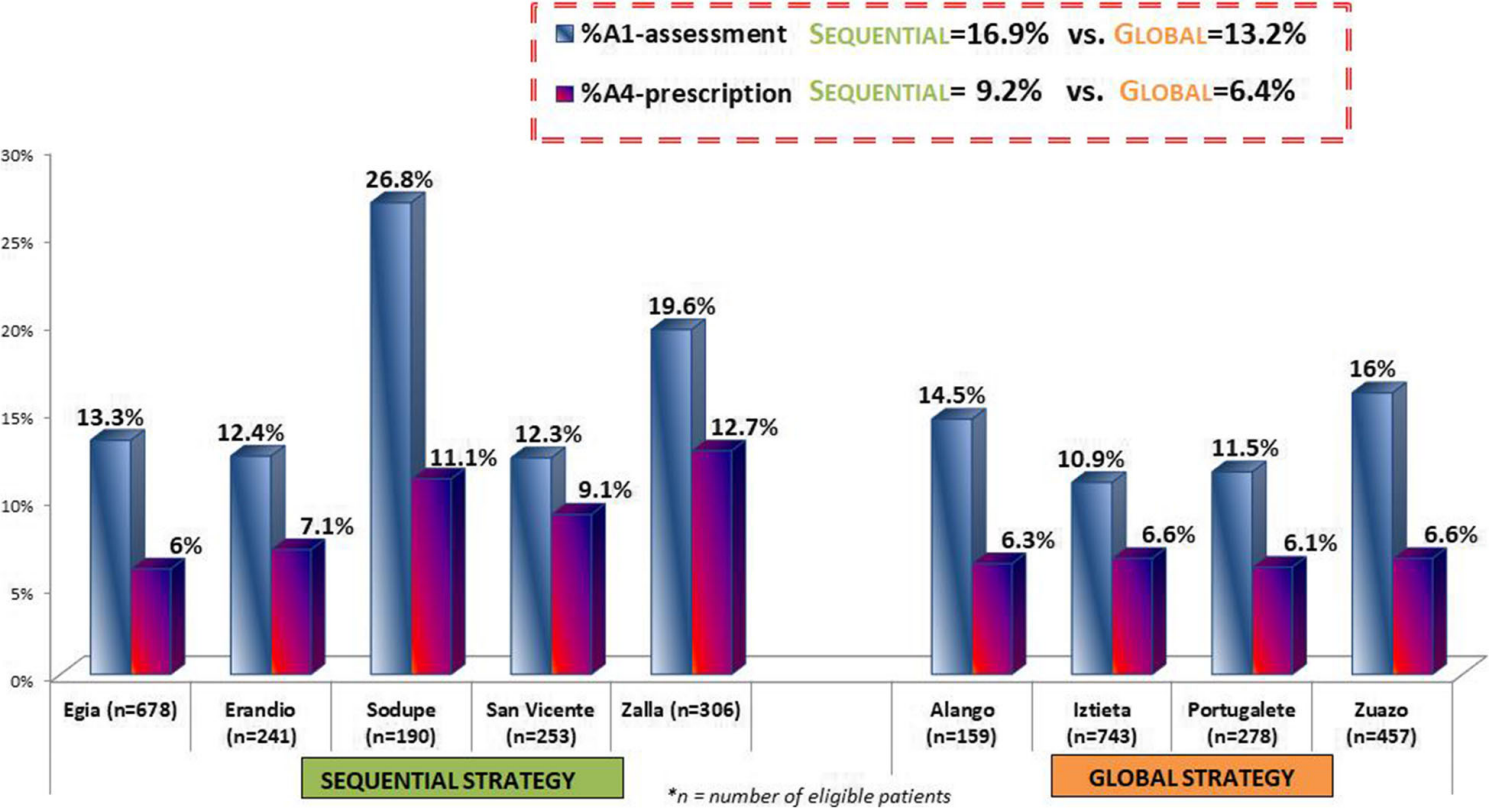
Reach and implementation of the PVS-PREDIAPS clinical intervention for type 2 diabetes prevention by center and comparison group. Note: Columns represent the percentage of non-diabetic patients aged ≥ 30 years with a known cardiovascular risk factor and an impaired fasting glucose level (≥ 110-125 md/dl) exposed to A1: assessment of healthy lifestyles or A4: prescription of lifestyle change with a personalized plan

Regarding the reach and implementation of the clinical intervention, 13.3% (*n* = 217; range, 11.4-16.3%) and 16.6% (*n* = 273; range, 11.9-29.3%) of the eligible patients were addressed by assessing at least one healthy lifestyle during the 12-month program implementation period in the global and sequential groups, respectively (Fig. 3). Thus, the overall reach of the intervention programs among all patients at high risk of developing T2D seen at least once in the nine participating centers was 15%. Among the eligible patients, a higher percentage of those in the sequential centers received personalized advice to change at least one habit (14.3%; *n* = 236 vs 11.3%; *n* = 183) in the global centers). The percentage of attending patients at risk of T2D receiving a personalized prescription of at least one lifestyle change (*N* = 251; 7.7%) was also higher in the sequential (8.6%; range 13.5-5.9%) than in the global group (6.8%; range 7.2-5.8%). Overall, 51% of all patients assessed then received a lifestyle prescription.

Regarding the specific lifestyle behaviors, the implementation of the intervention components was higher in relation to diet than physical activity (Table 1). Overall, 13.7% and 10.9% of patients underwent dietary assessment (being asked questions concerning daily servings of fruit and vegetables) and received a personalized advice to change diet, while these percentages were 10.9% and a 7.5% regarding physical activity assessment and advice, respectively. Notably, 6.8% of eligible patients were given personalized prescriptions to change diet, while only 1.7% received a prescription to increase physical activity. Except in the percentage of patients receiving a personalized prescription, the sequential group centers obtained higher figures in the rates of patients assessed and receiving advice for both lifestyles. On the other hand, for all reach and implementation indicators, within-group variability (that is, between centers) is higher among the centers belonging to the sequential group than the global group.

**Table 1:** Characteristics of eligible non-diabetic patients aged ≥ 30 years with a known cardiovascular risk factor and impaired fasting glucose level (≥ 110-125 md/dl) by center and comparison group

	Global group					Sequential group					
	PC1 *N* = 155	PC2 *N* = 456	PC3 *N* = 737	PC4 *N* = 278	***Total***	PC5 *N* = 228	PC6 *N* = 253	PC7 *N* = 665	PC8 *N* = 303	PC9 *N* = 188	***Total***
**Age, mean (sd)**	65.3 (13.7)	68.3 (12.5)	67.9 (12.2)	69.0 (11.6)	67.9 (12.4)	67.3 (13.5)	68.7 (13.2)	67.6 (13.2)	65.1 (12.4)	66.4 (12.6)	67.1 (13.0)
**Women** **n (%)**	73 (47.1%)	252 (55.3%)	385 (52.2%)	138 (49.6%)	848 (52.1%)	116 (48.7%)	122 (48.2%)	336 (50.5%)	143 (47.2%)	93 (49.5%)	810 (49.2%)
**BMI ≥ 25** **n (%)**	106 (68.4%)	286 (62.7%)	471 (63.9%)	177 (63.4%)	1040 (64.0%)	173 (72.7%)	166 (65.6%)	397 (59.7%)	231 (76.2%)	145 (77.1%)	1112 (67.5%)
**BMI ≥ 30** **n (%)**	65 (41.9%)	190 (41.7%)	272 (36.9%)	101 (36.3%)	628 (38.6%)	114 (47.9%)	105 (41.5%)	230 (34.6%)	145 (47.8%)	94 (50%)	688 (41.8%)
**SBP > 140** **n (%)**	61 (39.3%)	210 (46 %)	376 (51 %)	123 (44.2%)	770 (47.3%)	110 (46.2%)	131 (51.8%)	301 (45.3%)	143 (47.2%)	82 (43.6%)	767 (46.6%)
**DBP > 90** **n (%)**	38 (24.5%)	38 (8.3%)	82 (11.1%)	38 (13.7%)	196 (12%)	29 (12.2%)	34 (13.4%)	104 (15.6%)	49 (16.2%)	25 (13.3%)	241 (14.6%)
**Chol > 240** **n (%)**	28 (18.1%)	66 (14.5%)	156 (21.2%)	44 (15.8%)	294 (18.1%)	41 (17.3%)	36 (14.2%)	138 (20.7%)	50 (16.5%)	27 (14.4%)	292 (17.7%)
**Tri > 200** **n (%)**	20 (12.9%)	65 (14.2%)	80 (10.9%)	46 (16.5%)	211 (13%)	48 (20.2%)	48 (19%)	78 (11.7%)	41 (13.5%)	28 (14.9%)	243 (14.7%)
**Deprivation index**											
**High** **n (%)**	142 (91.6%)	49 (10.7%)	140 (19%)	105 (37.8%)	436 (26.8%)	13 (5.5%)	32 (12.6%)	226 (34%)	133 (43.9%)	35 (18.6%)	439 (26.6%)
**Medium** **n (%)**	9 (5.8%)	109 (23.9%)	219 (29.7%)	64 (23%)	401 (24.7%)	37 (15.5%)	65 (25.7%)	282 (42.4%)	96 (31.7%)	114 (60.6%)	594 (36.1%)
**Low** **n (%)**	4 (2.6%)	298 (65.3%)	378 (51.3%)	109 (39.2%)	789 (48.5%)	188 (79%)	156 (61.7%)	157 (23.6%)	74 (24.4%)	39 (20.7%)	614 (37.3%)
**Physical activity (PA)**											
**A1_PA** **n (%)**	19 (12.3%)	46 (10.1%)	44 (6%)	31 (11.1%)	140 (9%)	25 (10.5%)	24 (9.5%)	41 (6.2%)	49 (16.2%)	47 (25%)	186 (11.3%)
**A2_PA** **n (%)**	16 (10.3%)	28 (6.1%)	27 (3.7%)	25 (9%)	96 (5.9%)	21 (8.9%)	21 (8.2%)	23 (3.5%)	42 (13.9%)	42 (22.6%)	149 (9%)
**A4_PA** **n (%)**	5 (3.2%)	5 (1.9%)	12 (1.6%)	5 (1.8%)	27 (1.7%)	7 (3.1%)	7 (2.8%)	3 (0.4%)	5 (1.6%)	6 (3.2%)	28 (1.7%)
**Diet (DT)**											
**A1_DT** **n (%)**	23 (14.8%)	66 (14.5%)	78 (10.6%)	33 (11.9%)	200 (12.3%)	35 (14.7%)	26 (10.3%)	70 (10.5%)	65 (21.4%)	53 (28.2%)	249 (15.1%)
**A2_DT** **n (%)**	19 (12.3%)	52 (11.4%)	58 (7.9%)	27 (9.7%)	156 (9.6%)	29 (12.2%)	25 (9.9%)	48 (7.2%)	57 (18.8%)	43 (23.1%)	202 (12.3%)
**A4_DT** **n (%)**	8 (5.2%)	28 (6.1%)	46 (6.2%)	15 (5.4%)	97 (6%)	12 (5.3%)	21 (8.3 %)	37 (5.6%)	37 (12.2%)	20 (10.6%)	127 (7.7%)

Note: *BMI*, body mass index in kg/m^2^; *Chol*, cholesterol level in mg/dL; *DBP*, diastolic blood pressure in mmHg; *SBP*, systolic blood pressure in mmHg; *Tri*, triglyceride level in mg/dL

**Fig. 4:**
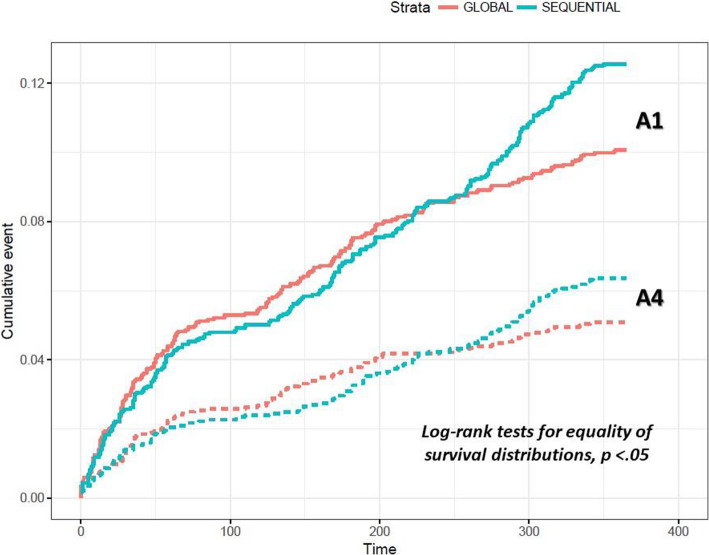
Cumulative exposure of patients to healthy lifestyle assessment and prescription over the 12-month program implementation period. Note: solid lines describe A1-assessment cumulative incidence; dotted lines describe A4-prescription cumulative incidence; cumulative event is measured in percentage; time is measured in days

A significant between-group difference was observed in the cumulative exposure of patients to healthy lifestyle assessment, advice, and prescription over the 12-month program implementation period (Log-rank test for equality of survival distribution, *p* < .05) (Fig. 4). When tested in an extended Cox model, a significant interaction was found between group and time, indicating that the proportionality assumption was not satisfied (group by time interaction for assessment, advice and prescription, p <.001). Specifically, during the first 8 months, the global group’s rates did not significantly differ from those obtained by the sequential group, while during the final 4 months, exposure to lifestyle assessment and to the prescription of lifestyle change was almost 2.5-fold (AHR for assessment, 2.61; 95% CI, 1.77-3.83) and 2.3-fold (AHR for prescription, 2.33; 95% CI, 1.38-3.94) more likely in sequential than in global centers, respectively. Exposure to lifestyle assessment and prescription were positively associated with the age of the patient and having obesity (BMI ≥ 30 kg/m^2^), but negatively associated with a high cholesterol level (≥ 240 mg/dl) and a high diastolic blood pressure (≥ 90 mmHg) (Table 2).

**Table 2:** Factors associated with exposure to the main intervention components: Assessment of lifestyle (A1), and prescription of lifestyle change (A4)

Variables		Adjusted hazard ratio (95% CI)	
		Assessed (A1)	Prescribed (A4)
**Age in years**	**≥ 75**	1.00	1.00
	**30-54**	3.14 (2.21-4.47)	3.33 (1.97-5.64)
	**55-64**	4.64 (3.40-6.33)	5.76 (3.63-9.12)
	**65-75**	3.96 (2.91-5.40)	4.51 (2.85-7.14)
**Sex**	**Female**	1.00	1.00
	**Male**	0.90 (0.75-1.08)	0.85 (0.66-1.09)
**Chol > 240**	**No**	1.00	1.00
	**Yes**	0.71 (0.55-0.92)	0.69 (0.45-0.99)
**DBP > 90**	**No**	1.00	1.00
	**Yes**	0.69 (0.52-0.92)	0.77 (0.53-1.13)
**BMI ≥ 30**	**No**	1.00	1.00
	**Yes**	1.28 (1.07-1.54)	1.35 (1.05-1.74)
**Group**	**Global**	1.00	1.00
**0-8 months**	**Sequential**	0.98 (0.79-1.20)	1.02 (0.76-1.36)
**9-12 months**	**Sequential**	2.61 (1.77-3.83)	2.33 (1.38-3.94)

Note: *BMI*, body mass index in kg/m^2^; *Chol*, cholesterol level in mg/dL; *DBP*, diastolic blood pressure in mmHg

Finally, regarding the spread of the healthy lifestyle promotion program actions in attending patients who do not meet the criteria for high risk of developing T2D (e.g., patients who are overweight or obese but have normal glucose levels), the absolute frequency of patients who have had their lifestyle assessed, been given advice, or been prescribed lifestyle change in all 9 collaborating centers has been 1551, 1246, and 476, respectively. Considering all ≥ 30-year-olds that attended their family physician or nurse at least once over the 12-month implementation period, the overall reach of the healthy lifestyle promotion program through the assessment of at least one lifestyle was 3.6%; while a 1.3% received a personalized prescription for changing at least one lifestyle behavior (2041 and 727 out of a total of 57,288 patients).

### Clinical outcomes in patients exposed to the intervention

A total of 432 patients ≥ 30 years old attending during the period of deployment of the program promoting healthy lifestyle habits had their lifestyle assessed using the PVS questionnaire at least 9 months after exposure to the intervention as part of routine care or at scheduled appointments (e.g., annual check-up for patients at risk of developing T2D) (see the characteristics of the sample Table 3 in Appendix). Of these, 206 had only received the assessment and advice components of the clinical intervention, while 226 patients had been given a personalized prescription of at least one healthy lifestyle habit, 86 concerning physical activity and 170 concerning diet.

**Fig. 5:**
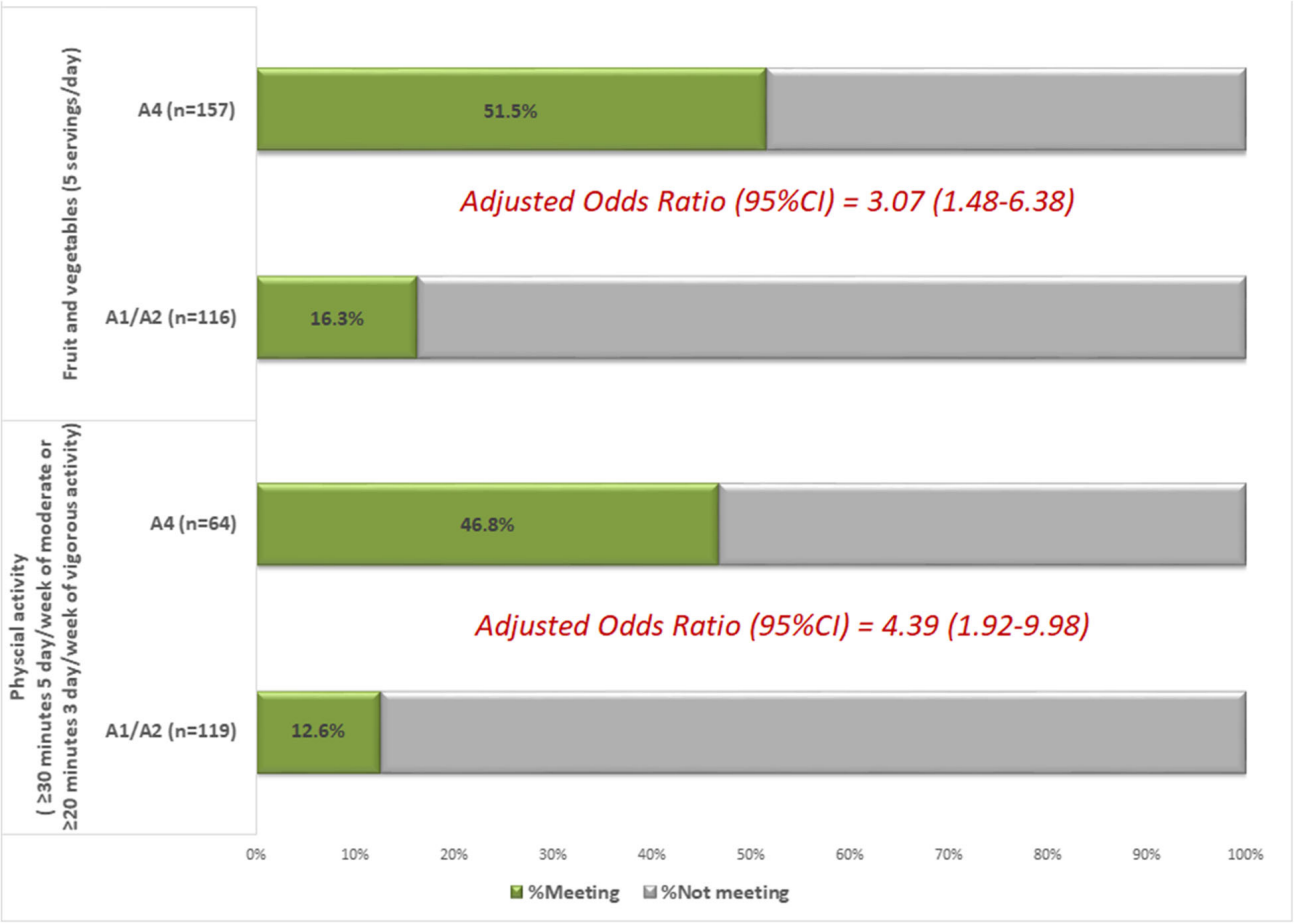
Effect of the personalized prescription in post-intervention lifestyle change in an incidental sample of patients exposed to the healthy lifestyle promotion program not meeting recommended levels at baseline

**Fig. 6:**
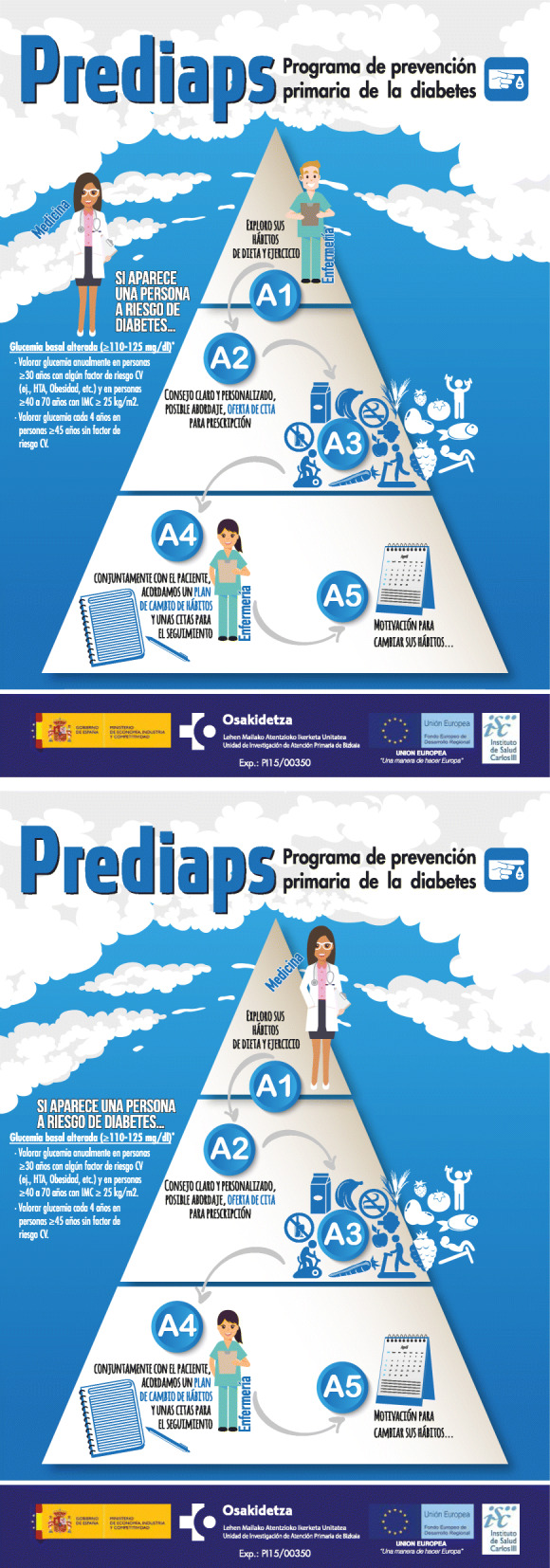
PREDIAPS clinical intervention delivery models

Regarding changes in level of physical activity, 46.8% of patients who did insufficient physical activity at baseline and received a physical activity prescription met the recommended levels of physical activity 12 months after the intervention (30 of the 64 who did not meet these levels at baseline and received the prescription) (see Fig. 5). Among patients who did insufficient physical activity at baseline and received assessment and advice, only 12.6% (15 out of 119) met the recommended levels at 12 months. According to the estimates from the adjusted multivariable model, after adjusting for the potential effect of confounders (e.g., age, sex, and BMI), the likelihood of meeting the recommended levels of physical activity was fourfold higher among patients given a prescription than among those assessed and advised (AOR, 4.39; 95% CI, 1.92-9.98). Concerning change in the adherence to recommendations on fruit and vegetable intake, among people who initially reported insufficient intake, 51.5% met this recommendation at 12 months among those prescribed a healthy diet and 16.3% met it among those assessed and advised. Having adjusted for third-level covariates, patients who received a prescription were 3 times more likely to meet the recommendations than those who received only assessment and advice (AOR, 3.07; 95% CI, 1.48-6.38). The effect of prescribing lifestyle change, both in terms of physical activity and diet, was not modified by the procedure of engagement in and execution of the strategy (p intervention component by group interaction, *p* > 0.05).

## Discussion

The objective of the implementation research project PREDIAPS [10] is to generate scientific knowledge on the effectiveness of different procedures for engaging health professionals and executing a facilitated collaborative modeling approach for incorporating an evidence-based T2D prevention program into the portfolio of services offered by PC centers in a feasible and sustainable manner, by maximizing its reach and efficiency among patients who would potentially benefit from the program. The results of the study show that, in general, clinical practice in promoting healthy lifestyle habits for the prevention of T2D in high-risk patients from the 9 participating centers changed significantly. According to the scientific evidence, to successfully reduce the risk of developing T2D in high-risk patients, we need to provide an intensive intervention seeking to increase their levels of physical activity and/or improve their diet [1–5]. Currently, in the real context of the portfolio of services for the prevention of T2D in our health system (Osakidetza), only 30% of patients at a high risk of developing this condition receive some type of intervention focused on assessing their lifestyle (e.g., “Do you do regular exercise? (yes or no)”) and general preventive advice. In the framework of the PREDIAPS project, health centers have reached 15% of individuals at high risk of developing T2D over 1 year, with a more valid assessment of lifestyle (e.g., “minutes/week of physical activity at a moderate-to-vigorous intensity;” “adherence to recommended levels of physical activity”), some centers reaching as much as 30% of this at-risk population. Furthermore, PC teams succeeded in prescribing lifestyle change with a personalized plan (an intensive intervention that is effective for the prevention of T2D) to 7.5% of the population seeking medical attention in the participating centers ≥ 30 years of age and at high risk of developing T2D, some centers even reaching as much as 13.5% of this population.

These findings are particularly notable if we compare them with those of other initiatives for the translation of recommended interventions for T2D prevention to routine practice. A study carried out in two healthcare systems delivering most of the clinical care in a state in the USA [32], seeking to assess the reach of screening and evidence-based certified intensive interventions for T2D prevention (Center for Disease Control and Prevention-recognized Diabetes Prevention Programs), estimated that very few patients found to be at high risk of developing this disease participated in a prevention program (0.52%; range 0-3.53%). The authors concluded that there was a need to use implementation strategies to improve the rates of referral. Other initiatives that have sought to use specific implementation strategies to maximize the adoption and deployment of recommended interventions for T2D prevention have obtained somewhat better outcomes. In a study by Keck et al. [33], with implementation activities involving targeted clinician education, a prediabetes clinician champion, and a custom electronic health record report identifying patients with prediabetes, centers succeeded in referring 6.9% of patients with prediabetes to Diabetes Prevention Programs. In the context of the Spanish Health System, the Diabetes Prevention-Transferring findings from European research to society project [9] found that the intervention was delivered to 80% of patients recruited and identified as at high risk (n = 1819, from 83 PC centers), but we do not know the reach among all users of participating centers at high risk of developing T2D.

The main challenge of IPC is how to achieve a functional and effective collaborative interaction, respecting the professional differences in terms of identity, values, power, and competencies of the involved professional specialties [11, 25, 34]. Consequently, in our opinion, the most important finding of our study was that a sequential facilitated collaborative process, focused first on nurses who subsequently seek the pragmatic cooperation of GPs to model and tailor the recommended intervention for T2D prevention in PC, showed the best implementation results. Specifically, 12 months after incorporating the program into the daily practice of the centers, the cumulative percentage of patients exposed to the different components of the intervention was higher in the centers that used this sequential strategy. Indeed, in two of the sequential group centers almost or more than 20% of users who were seen and identified as at high risk of developing T2D had lifestyle assessments completed. Further, over the 12-month program implementation period, the trajectories of both the overall reach of the program (through the assessment of at least one lifestyle) and the implementation of the main intervention component to prevent T2D (the prescription of lifestyle change with a personalized plan to change at least one lifestyle) differed significantly between the groups compared. The difference was most notable at 8 months, coinciding with the end of the summer holidays for most health professionals (August), the speed of implementing the program, measured in terms of monthly rate, having decreased before this point and more markedly so in the global than sequential group centers.

A plausible explanation of this finding, our current hypothesis to account for our results, may lie in the theoretical assumptions behind the planned and tested differentiation of procedures for involving physicians and nurses and performing the PREDIAPS implementation strategy to optimize DT2 prevention through health promotion, namely, that there is a synergy between collaboration and autonomy, both being important concomitant elements influencing interprofessional team functioning. In short, some authors [25, 34] have argued that autonomy may be an important element of successful IPC and that concerns about limited autonomy or inability of individuals to practice to their full scope have shown to impede the implementation of IPC [11, 35], or even more, increase physicians’ workload and consequently negatively influence collaborative practice [36]. Moreover, some authors argue that empowering team members to develop autonomy, while still having interchangeable responsibilities, interactions, and knowledge exchanges with other professions, can enhance collaborative interactions [34]. Within the PREDIAPS study, in the group that used a global strategy, both doctors and nurses are involved from the outset in carrying out the implementation strategy and its actions, seeking to achieve adoption and deployment of the clinical intervention to the context of individual centers. In contrast, the sequential strategy respects the autonomy of the professional groups while trying to generate multi-professional pragmatic cooperation, being in this way less complex, more flexible, and adaptable to the real context of each center, and probably generating fewer conflicts between professional categories. In addition, it is rooted in nurses, which on the one hand, enhance the likelihood of nurses taking the responsibility for executing the clinical intervention from the beginning, and on the other hand, reinforce the their protagonist role in the process of change and the planning of the intervention program.

Regarding the clinical outcome measures, lifestyle changes among patients exposed to the clinical intervention, the analysis of potential effectiveness based on the observational analysis of a subsample of patients seems to indicate that the intervention, and specifically the prescription of lifestyle change, is associated with changes in adherence to recommended levels of physical activity and fruit and vegetable intake. The percentage of patients who change their lifestyle habits is higher among individuals who do than those who do not receive an intervention for prescribing lifestyle change with a plan. Specifically, the likelihood of meeting the recommended levels among those exposed to this type of intervention is four- and threefold higher for physical activity and fruit and vegetable intake, respectively. Nonetheless, given the limitations of this part of our analysis, it being carried out in a subsample of patients exposed, we are unable to conclude that the changes observed are solely attributable to the intervention. On the other hand, we can state that, unlike our findings regarding the implementation outcomes, the effect of the clinical intervention on patients in terms of the modification of health-related behaviors did not differ as a function of the procedure for engaging health professionals and executing the PREDIAPS implementation strategy.

We consider that our study has yielded significant findings regarding the potential effect on change in habits of the prescription of lifestyle change with a personalized plan for increasing the level of physical activity (type, frequency, duration, intensity, and progression) or improving diet (in accordance with recommendations on a Mediterranean-type healthy diet) and their follow-up in the context of routine PC practice. Increasing individuals’ level of physical activity and improving their diet, together with weight loss, are the most important changes in lifestyle associated with reducing the risk of developing T2D [1–4]. Lifestyle changes following interventions promoting healthy habits led by nurses, mainly focusing on healthy physical activity and dietary habits, in patients with a high risk of developing T2D, in the routine context of PC, have shown to reduce the risk of developing T2D by almost a third [7, 37]. Moreover, the effects of diet and physical activity promotion programs to prevent T2D seem to be long lasting [38] and are cost-effective in at-risk groups [39].

Our study has several limitations. First, the relatively small number of centers included together with the procedure for their selection has implications for the generalizability of our findings. Centers were identified upon the suggestion of the medical management offices of the collaborating integrated health organizations, this resulting in a convenience sample, not representative of all PC centers in our health service. Although collaborating centers are apparently diverse (in terms of size and composition, and socioeconomic status of their catchment populations, among other characteristics), missed information regarding other relevant contextual factors may hamper the interpretation of our results. The second main limitation is related to the clinical outcome measure used to assess the potential effectiveness of the clinical intervention in changing lifestyles. In short, evaluation of potential effectiveness has been performed with an incidental sample of patients exposed to the clinical intervention who attended to follow-up visits as part of receiving routine care or for the annual check-ups recommended for the primary prevention of T2D in high-risk patients. As selection bias and other confounding factors may have affected our analysis of clinical outcomes, the results must be considered observational in nature, meaning we cannot attribute the results observed to the clinical intervention alone. In addition, the limited number of patients finally included for the potential effectiveness analyses may affect the precision of estimated effects. The main strength of the study is that it has been conducted under real-world conditions in PC without altering the usual working conditions. Another strength is the quality of data related to implementation results as these were recorded in and subsequently retrieved from an established electronic clinical record system.

## Conclusion

The PREDIAPS implementation strategy succeeds in changing and optimizing clinical practice and its organization, involving professionals themselves in the feasible, effective, and sustainable translation of interventions with proven efficacy for preventing T2D to practice, and thereby improving the provision of care by our healthcare professionals. This project helps to deepen our knowledge concerning organizational models and strategies suitable for favoring collaboration through the creation of communities of practice, focused on collaborative planning and the redefinition of roles, distribution of tasks, and optimization of care delivery and services. Specifically, the results of this study suggest that, in areas in which nurses play a leading role, that is, disease prevention and health promotion, a procedure for promoting IPC that safeguards the autonomy and strengthens the responsibility of this profession achieves better outcomes in terms of adoption and deployment of evidence-based interventions for the prevention of T2D at the PC level. The present study therefore contributes with scientific knowledge in the field of implementation science about the effectiveness of different strategies and procedures to create functional and effective collaborative interprofessional teams within PHC, in a global context of high workload and saturation, where different health professionals provide services with marked class differentiation, both in terms of identity and competence. All these with a view of maximizing their potential to improve or change organizational performance and the provision of evidence-based preventive healthcare services.

## Data Availability

Since data supporting the present study will mostly concern to process data and reports from specific professionals of the Basque Health Service-Osakidetza, it will be only shared upon justified request to the study guarantors.

## Appendix

**Table 3:** Characteristics of the sample (*n* = 432) of patients ≥ 30 years of age seen during the implementation period and exposed to components of the intervention by group and level of exposure (A1/A2: assessment and advice; A4: prescription of lifestyle change) used for the analysis of clinical outcomes

	Global			Sequential		
	A1/A2 *N* = 72	A4 *N* = 103	Total	A1/A2 *N* = 134	A4 *N* = 123	Total
**Age, mean (sd)**	68.0 (9.5)	62.7 (10.8)	64.9 (10.6)	67.6 (10.9)	61.2 (10.4)	64.5 (11.1)
**N (%) women**	29 (40.3%)	64 (62.1%)	93 (53.1%)	61 (45.5%)	63 (51.2%)	124 (48.2%)
**≥ 30 years + CVRF** **n (%)**	53 (73.6%)	83 (80.6%)	136 (77.7)	105 (78.4%)	104 (84.5%)	209 (81.3%)
**IFG** **n (%)**	13 (18.1%)	39 (37.9%)	52 (29.7%)	25 (18.7%)	47 (38.2%)	72 (28%)
**Diabetic** **n (%)**	19 (26.4%)	13 (12.6%)	32 (18.3%)	47 (35.1%)	17 (13.8%)	64 (24.9%)
**BMI ≥ 25** **n (%)**	58 (80.6%)	80 (77.7%)	138 (78.9%)	119 (88.8%)	102 (82.9%)	221 (86%)
**BMI ≥ 30** **n (%)**	23 (31.9%)	45 (43.7%)	68 (38.9%)	64 (47.8%)	66 (53.7%)	130 (50.6%)
**DBP > 140** **n (%)**	27 (37.5%)	36 (34.9%)	63 (36%)	51 (38.1%)	41 (33.3%)	92 (35.8%)
**DBP > 90** **n (%)**	4 (5.6%)	16 (15.5%)	20 (11.4%)	12 (9%)	16 (13%)	28 (10.9%)
**Chol > 240** **n (%)**	7 (9.7%)	10 (9.7%)	17 (9.7%)	14 (10.4%)	14 (11.4%)	28 (10.9%)
**Tri > 200** **n (%)**	5 (6.9%)	10 (9.7%)	15 (8.6%)	14 (10.4%)	15 (12.2%)	29 (11.3%)
**Meet recommended level of physical activity at baseline n (%)**	33 (45.8%)	33 (32%)	66 (37.7%)	51 (38.1%)	41 (33.3%)	92 (35.8%)
**Meet recommended intake of fruit/vegetables at baseline n (%)**	27 (37.5%)	21 (20.4%)	48 (27.4%)	53 (39.5%)	11 (8.9%)	64 (24.9%)

Note: *BMI*, body mass index in kg/m^2^; *≥ 30 years + CVRF*, 30 years of age or more with cardiovascular risk factors; *Chol*, cholesterol level in mg/dL; *DBP*, diastolic blood pressure in mmHg; *IFG*, impaired fasting glucose level; *SBP*, systolic blood pressure in mmHg; *Tri*, triglyceride level in mg/dL
